# A Focus on the Functions of Area 25

**DOI:** 10.3390/brainsci9060129

**Published:** 2019-06-03

**Authors:** Laith Alexander, Hannah F. Clarke, Angela C. Roberts

**Affiliations:** 1 Department of Physiology, Development and Neuroscience, University of Cambridge, Cambridge CB2 3DY, UK; la326@cam.ac.uk; 2 Behavioural and Clinical Neuroscience Institute, Department of Psychology, University of Cambridge, Cambridge CB2 3EB, UK

**Keywords:** area 25, infralimbic, autonomic, emotion, anhedonia, negative affect, anticipatory arousal

## Abstract

Subcallosal area 25 is one of the least understood regions of the anterior cingulate cortex, but activity in this area is emerging as a crucial correlate of mood and affective disorder symptomatology. The cortical and subcortical connectivity of area 25 suggests it may act as an interface between the bioregulatory and emotional states that are aberrant in disorders such as depression. However, evidence for such a role is limited because of uncertainty over the functional homologue of area 25 in rodents, which hinders cross-species translation. This emphasizes the need for causal manipulations in monkeys in which area 25, and the prefrontal and cingulate regions in which it is embedded, resemble those of humans more than rodents. In this review, we consider physiological and behavioral evidence from non-pathological and pathological studies in humans and from manipulations of area 25 in monkeys and its putative homologue, the infralimbic cortex (IL), in rodents. We highlight the similarities between area 25 function in monkeys and IL function in rodents with respect to the regulation of reward-driven responses, but also the apparent inconsistencies in the regulation of threat responses, not only between the rodent and monkey literatures, but also within the rodent literature. Overall, we provide evidence for a causal role of area 25 in both the enhanced negative affect and decreased positive affect that is characteristic of affective disorders, and the cardiovascular and endocrine perturbations that accompany these mood changes. We end with a brief consideration of how future studies should be tailored to best translate these findings into the clinic.

## 1. Introduction

**Figure 1:**
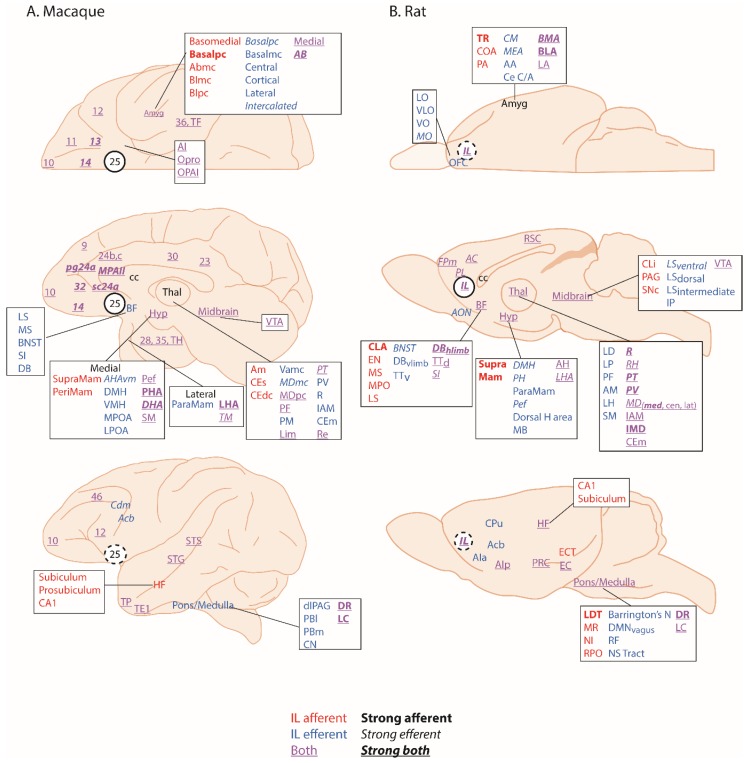
The connectivity of area 25 in macaques and the infralimbic cortex (IL) in rodents. In the macaque (**A**) area 25 has widespread efferent and afferent connections with many cortical and subcortical regions that are comparable to the efferent and afferent connections of IL in the rat (**B**). Afferents and efferents are depicted superimposed on orbital, medial and lateral views of the macaque and rat brains based on anterograde and retrograde tracing studies. Macaque: [1,4,5,6,7,8,9,10,11,12,13,14,15]. Rat: [4,6,9]. Abbreviations: AC, Anterior Cingulate; Acb, Accumbens; AIa/p, Agranular Insula anterior/posterior; Amyg, Amygdala (AA, anterior amygdala; AB, Accessory Basal; BMA, basomedial; BLA, basolateral; Bpc/mc, Basal parvicellular/magnocellular; CE, CM, centromedial; central; COA, cortical; LA, lateral; MEA, medial; PA, posterior; TR, Amygdalo-piriform transition zone); AON, Anterior Olfactory Nucleus; BF, Basal Forebrain (BNST, Bed Nucleus of the Stria Terminalis; DB, Diagonal Band of Broca; EN, endopiriform nucleus; LS, Lateral Septum; MPO, Medial Preoptic Area MS, Medial Septum; SI, Substantia Innominata; TTv/d, ventral/dorsal Taenia Tecta); cc, Corpus Callosum; Cdm, medial Caudate; CLA, Claustrum; CLi, Central linear nucleus; CPu, CaudatePutamen; EC, Entorhinal Cortex; ECT, Ectorhinal Cortex; FPm, Frontal Polar Cortex, medial; HF, Hippocampal Formation, Hyp, Hypothalamus (AH, anterior nucleus; AHAvm, Anterior Hypothalamic area, ventromedial; DHA, Dorsal Hypothalamic area; DMH, Dorsomedial, LHA, Lateral Hypothalamic area; LPOA, lateral Preoptic area; MB, Mammillary bodies; MPOA, medial Preoptic area; Pef, Perifornical; PH, Posterior nucleus; PHA, Posterior Hypothalamic area; PeriMam, Peri-mammillary; SupraMam, Supra-mammillary; TM, Tubero-mammillary; ParaMam, Paramammillary); IL, Infralimbic;; IP, interpeduncular nucleus; OFC, Orbitofrontal cortex (LO, lateral orbital; VLO, ventrolateral orbital; VO, ventral orbital; MO, medial orbital); Opro, Orbital proisocortex; OPAI, Orbital periallocortex; Pons/Medulla (Barrington’s N, Barrington’s Nucleus; CN, Cuneiform nucleus; dlPAG, dorsolateral Periaqueductal Grey; DMNvagus, Dorsal Motor Nucleus of Vagus; DR, Dorsal Raphe; LC, Locus Coeruleus; LDT, Laterodorsal tegmental nucleus; MR, Median Raphe; NI, Nucleus Incertus; NSTract, Nucleus of the Solitary Tract; PBl/m, Parabrachial lateral/medial; RF, Reticular Formation); pg, perigenual; PL, Prelimbic; PRC, Perirhinal Cortex; RPO, Nucleus pontis oralis; RSC, Retrosplenial Cortex; SNc, Substantia nigra pars compacta; Thalamus: (Am, medial Anterior; CEs/m/dc, Central superior/medial/ densocellular; IAM, Interanteriomedial; IMD, Intermediodorsal; LD, Lateral dorsal; LH, Lateral Habenula; Lim, Limitans; LP, Lateral posterior; MDpc/mc, Mediodorsal parvocellular.magnocellular; PF, parafascicular; PM, medial Pulvinar; PT, Parataenial; PV, Paraventricular, R, Reuniens; RH, Rhomboid; sc, subcallosal; SM, Submedial; Vamc, Ventral Anterior magnocellular); STG, Superior Temporal Gyrus, STS, Superior Temporal Sulcus; TEI, TF, TH, TP, Temporal pole; VTA, Ventral Tegmental Area; Brodmann’s Areas, 9, 10, 11, 12, 13, 14, 23, 24, 25, 28, 30, 32, 35, 36, 46, TE1, TF, TH. Nomenclature of prefrontal cortex parcellation in macaque based on [15].

Area 25 is found within the subcallosal cortex and is one of the least understood regions of the anterior cingulate cortex (ACC). It is variably included with adjacent regions of the subcallosal zone (scACC), also referred to as subgenual, and more broadly, the ventromedial prefrontal cortex (vmPFC), which includes medial PFC regions anterior to the genu of the corpus callosum. Although there is variation in the precise extent of area 25 within current architectonic maps of this area, particularly in the macaque [1,2], there is consensus with respect to its extreme caudal position within the subcallosal region, lying adjacent to the lateral septum of the basal forebrain. Where maps in the macaque differ is with respect to whether area 25 extends onto the orbital surface or not. Area 25 is characterized as agranular cortex in humans, with no identified granular layer IV [3], and dysgranular cortex in monkeys [1] with a very thin granular layer IV, anteriorly. The overall layering within area 25 is relatively undifferentiated, with fusion of layers II and III into a broad supragranular layer and fusion of layers V and VI into a much narrower, but dense infragranular layer. Based on tracing studies in rhesus macaques (Figure 1A), area 25 is densely connected with neighboring ventromedial and posterior orbitofrontal cortex (OFC), involved in affective evaluation and a moderate pathway linking it to fronto-polar area 10 and cognition. Outside of the PFC, area 25 has strong connections with the interoceptive regions of the anterior insula, the medial temporal lobe memory system, the auditory association cortex in the superior temporal gyrus and the multimodal superior temporal sulcus [1]. As described in [1], the propensity of area 25 projections to originate in deep layers and end within superficial layers of eulaminate brain regions is a pattern normally associated with feedback organization. However, its connectivity with agranular regions, including neighboring posterior OFC, vmPFC, medial temporal lobe (MTL) and anterior insula, appears feedforward in nature, suggesting that activity is initiated in area 25. Subcortically, area 25 has by far the strongest reciprocal connectivity with the amygdala compared to all other prefrontal regions. It also has dense projections to the ventral striatum, a number of nuclei within the hypothalamus (including the preoptic area, lateral hypothalamus and dorsomedial hypothalamus), bed nucleus of the stria terminalis, medial septum, diagonal band of Broca and substantia innominata. In the brainstem there are also projections to the monoamine systems as well as the periaqueductal grey and parabrachial nucleus [1,2]. This connectivity pattern positions area 25 at the intersection between emotion, visceromotor function and memory. 

According to Vogt [16], area 25 can be identified across humans, monkeys, rats and mice which should facilitate inter-species translation of findings across experimental studies. However, structural homology does not necessarily equate to functional homology. In rodents, the area identified as area 25 by Vogt, is commonly called infralimbic cortex (IL) and so will be referred to as IL in this review. As would be expected if primate area 25 and rodent IL are homologous, IL projects to many of the same cortical and subcortical projection areas linked to emotion, visceromotor control and memory as area 25 in the macaque [4] (Figure 1B). Certainly, Haber and colleagues have confirmed the similarity of the IL-25-striatal projection pattern across rats and macaques [5]. In contrast, Barbas and colleagues [17] suggest that IL‒amygdala projections of the rat more closely resemble the posterior OFC‒amygdala projections of a macaque because of the similarity of the projections onto the GABAergic inhibitory intercalated masses of the amygdala. Notably absent in rats [4,6] are connections with auditory association and polymodal sensory association cortices that have been described in macaques. Conversely, in rats, IL projects to the accessory olfactory nucleus involved in olfactory processing and the Nucleus of the Solitary Tract and other autonomic effector regions in the brainstem, which are not innervated directly by the primate area 25 [1]. Moreover, as will be seen later in the descriptions of functional effects of the rodent IL compared to the monkey area 25, potential differences do emerge, suggesting that the assumption of functional homology between these regions may be premature. Another issue hampering translation is that, often when describing activation foci within the subcallosal zone, human neuroimaging studies do not differentiate between the distinct cytoarchitectonic regions present within this zone [18]. Moreover, in some cases, the term vmPFC is used instead to refer to an even broader area that not only includes the subcallosal cortex, but extends rostrally into ventromedial regions lying in front of the genu [19]. As a consequence, activation loci that truly include area 25 are less evident in the human neuroimaging literature [20], highlighting why current understanding of the functions of this region is particularly dependent upon neurobiological studies targeting area 25 in monkeys. By careful comparison with studies of IL in rodents we can begin to piece together the functions of this region, determine the extent of functional homology, and inform future studies in humans.

## 2. Physiological Function and the Subcallosal Zone

A prominent function of area 25, and one that is perhaps key to our overall understanding of this subcallosal region, is its involvement in visceral control and feedback mechanisms involving cardiovascular, endocrine and immune systems. Area 25 has been implicated in the regulation of autonomic (particularly cardiovascular) and endocrine functions in studies of non-human primates and humans, and significant insight has also been gleaned from studies in rodents exploring the anatomical connectivity and functional importance of its putative homologue, IL, as described in detail below. When reviewing this literature two factors should be taken into account. First, whether the study has employed an awake or anesthetized preparation, since anesthesia is known to alter cardiovascular activity [21]. Second, how the cortex has been manipulated. Several early functional studies employed electrical stimulation [22] and the frequency of stimulation, together with pulse duration, can result in differing magnitudes of effects and activate adjacent fiber pathways [23]. Furthermore, whether the impact of electrical stimulation is analogous to ‘activating’ or ‘inhibiting’ a brain region is not always known. 

### 2.1. Area 25 and Cardiovascular Function

In humans, despite the strong connections between area 25 and autonomic control centers, neuroimaging studies investigating heart rate (HR) and blood pressure (BP) regulation have consistently implicated more dorsal and perigenual (pg)ACC regions rather than more ventral area 25 [24,25,26,27,28]. However, investigations of the dynamic adjustment of HR, using measures of heart rate variability, do implicate area 25 activity across cognitive, motor and affective manipulations [29]. In particular, activity in the caudal regions of human vmPFC, that included area 25, correlated positively with vagal tone whilst shifting between affective states [30]. This linked area 25 and vmPFC BOLD activity patterns directly with high frequency band components of heart rate variability, thought to reflect parasympathetic activity [31]. Thus, in particular, area 25 may modulate parasympathetic output [32,33].

**Figure 2:**
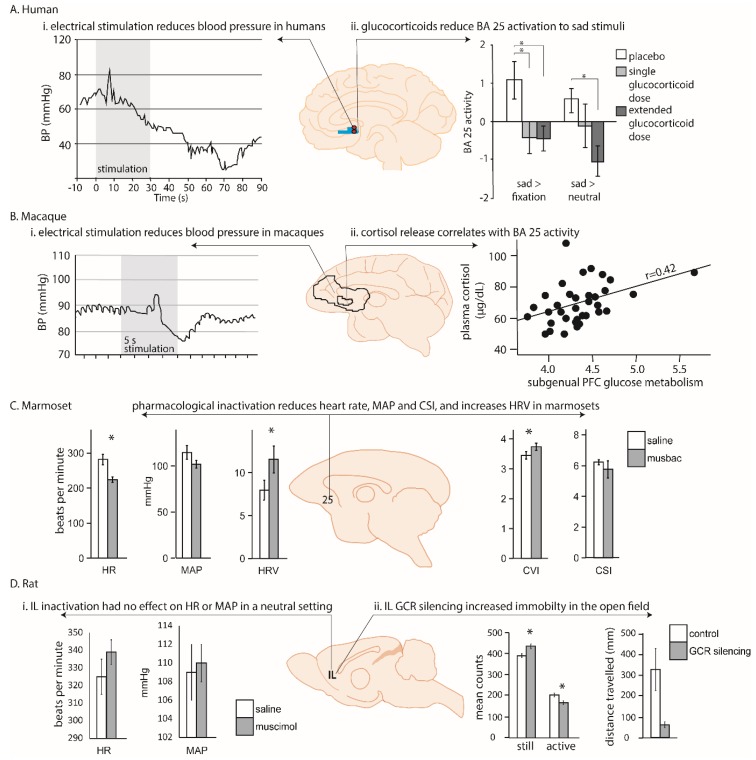
Examples of the relationship between cardiovascular and endocrine responsivity and area 25/infralimbic (IL) activity in emotional and non-emotional conditions in human, macaque, marmoset and rat. (**A**) In humans, (**i**) stimulation of area 25 via deep brain stimulation (red circles indicate stimulation loci) causes a brief hypertension followed by pronounced hypotension in anesthetized humans [34] while (**ii**) the administration of peripheral cortisol decreases area 25 activation to sad stimuli (brain region shaded blue) in the absence of any stressors [36]. (**B**) In macaques (**i**) stimulation of area 25 in anesthetized animals caused a brief hypertension followed by pronounced hypotension [22], similar to humans, and (**ii**) cortisol release correlates with ventromedial prefrontal cortex (vmPFC) activity during neutral conditions (large demarcated area), and specifically with area 25 during both stressful and neutral conditions (small demarcated area; [37]). (**C**) In marmosets, pharmacological inactivation of area 25 with GABA A and B agonists (muscimol and baclofen; ‘musbac’) in a neutral condition caused a reduction in heart rate (HR) and mean arterial blood pressure (MAP) that was accompanied by an increase in heart rate variability (HRV). Subdivision of the cardiac vagal and cardiac sympathetic indices (CVI and CSI) revealed that this HRV change was caused by a selective increase in the parasympathetic CVI [38]. (**D**) In rats (**i**) inactivation of IL had no effect on HR and MAP [39], unlike humans and non-human primates, but (**ii**) selective glucocorticoid receptor (GCR) silencing within the IL reduced activity within the open field test indicating that cortisol can modulate IL’s impact on emotional behavior [40]. Thus, whereas manipulation of area 25 in a neutral setting consistently modulates cardiovascular function in humans and non-human primates, such changes are not apparent after IL manipulation in the rat. However, glucocorticoids appear to modulate negative emotion in both area 25 and IL indicating similarities in some functional domains, but not others.

More rarely, modulation of cardiovascular function has also been observed following deep brain stimulation of the human vmPFC, including area 25, in patients undergoing electrode implantation as a prelude to surgery to relieve epilepsy [34]. In the four patients with electrodes within scACC, stimulation produced consistent and striking hypotensive changes; specifically, a reduction in systolic BP with more variable changes in diastolic BP. Hypotensive effects were substantially greater in those patients with a more caudal placement in area 25 compared to a more rostral placement (within area 14; Figure 2Ai). However this interpretation is confounded by differences in the laterality of the hemisphere stimulated, and uncertainty over whether the neurophysiological consequence of deep brain stimulation is excitation, inhibition, or a more generalized disruption [35]. Thus, although scACC/25 is implicated in autonomic, particularly parasympathetic, regulation, the precise role area 25 plays in humans is still unclear. 

Consistent with humans, anatomical tracing of area 25 connectivity in macaque monkeys shows dense projections to the hypothalamic autonomic nuclei which then project to the nucleus of the solitary tract and spinal autonomic centers [41]. Regions of non-human primate vmPFC, including area 25, also diffusely innervate multiple amygdala nuclei, meaning there is dual access to an emotional-visceral motor system [42,43]. Consistent with these anatomical connections, selective manipulations of non-human primate area 25 have shown the importance of this area in contributing to autonomic regulation.

Early functional work, much of which was carried out in macaques, largely focused on determining the contributions of the cingulate gyrus to cardiovascular regulation, rather than the involvement of area 25 specifically [44]. Nevertheless, evidence for a role of ventral subregions came when Kaada and colleagues applied electrical stimulation to regions of pgACC and scACC in anesthetized macaques [22]. Stimulation throughout these regions induced cardiovascular changes, with the most prominent cardiovascular change observed in ‘posterior subcallosal cortex,’ corresponding to area 25. In addition to having a respiratory effect, application of electrical current in this region produced a BP response, characterized by a transient hypertension followed by a more prolonged—but still short-lived—refractory hypotension (Figure 2Bi). These data support the findings in humans, in which hypotension was observed following deep brain stimulation in caudal vmPFC regions [34]. However, apart from the observation that enhanced activity within scACC/25 is seen during vegetative states such as sleeping (which potentially also reflects an influence on parasympathetic activity [45]), there has been little further electrophysiological investigation of the role of the primate vmPFC in autonomic regulation.

More recently, targeted pharmacological manipulations within the non-human primate vmPFC have specifically dissected out area 25’s role in autonomic regulation [38]. Inactivation of area 25 (using a cocktail of GABA A and B agonists) in marmosets, New World monkeys, whilst in an emotionally neutral, quiet resting state, was found to have profound effects on cardiovascular activity, reducing HR and BP and increasing heart rate variability. When effects on heart rate variability were fractionated into vagal (parasympathetic) and sympathetic contributions, area 25 inactivation selectively increased cardiac vagal tone. These effects should be contrasted with the very limited effects on baseline cardiovascular activity, in the form of a modest increase in BP, which followed inactivation of area 32. Area 25 inactivation also reduced the learned HR increases associated with a stressful outcome, while area 32 inactivation elevated them [38], and area 25, but not area 32, activity mediated the normalization effects of hippocampal activation on the autonomic correlates of high-trait anxious responses [46]. Together, these pharmacological studies suggest that non-human primate area 25 has a critical causal role in modulating activity within a central autonomic network during both neutral and emotional states.

These findings do have some apparent similarity to a large body of work in rodents that supports a role for rodent vmPFC, including IL, in the regulation of cardiovascular function. Like in humans and non-human primates, the anatomical connectivity of IL points to a role in autonomic regulation. The IL projects to many autonomic control regions (the hypothalamus, amygdala, insula and periaqueductal gray [4,23]) which in turn project to the autonomic effector regions in the brainstem (Figure 1B). The IL and ventral aspects of the PL also project directly to these brainstem systems including the nucleus of the solitary tract [47] and the spinal cord (intermediolateral nucleus) [48]. These direct projections have led some researchers to coin the term ‘visceral motor cortex’ for these regions [23,47].

The anatomy is supported by functional studies of cardiovascular regulation which clearly demonstrate that IL manipulations can alter cardiovascular function. Nevertheless, it is difficult to compare these findings with those of non-human primates due to differences in the pharmacological compounds used, and variations in behavioral paradigms and types of stressor. Furthermore, only a few studies have investigated the consequences of IL manipulation in baseline, emotionally neutral conditions. Thus, inactivation of IL with a localized muscimol microinfusion had no effect on cardiovascular control during baseline conditions [39], whilst IL disinhibition with bicuculline increased respiratory and cardiac outflow [49]. In stressful situations the IL does appear to regulate cardiovascular responses, but in a stressor-specific manner [50]. Cobalt chloride injection in the IL (which silences inputs and outputs and ‘inactivates’ the brain region) reduces tachycardia associated with restraint stress [51]. In contrast, IL inactivation with muscimol did not alter the cardiovascular responses induced by air puff stress, indicating that there are either functional differences in the mechanism of inactivation, or behavioral differences as a consequence of different stressors [39]. Different again, activation of IL with the excitatory amino acid, *N*-Methyl-d-Aspartate (NMDA) did decrease the HR and BP responses induced by the same air puff [39]. Although both these stressors are unconditioned, air puff is considered a milder stressor compared to restraint stress. 

In studies that have investigated the involvement of the IL in regulating conditioned (learned) cardiovascular responses it has been shown that excitotoxic lesions primarily targeting the IL (with variable involvement of the more dorsal prelimbic [PL] subregion) decreased HR responses to a tone predicting shock [52]. This is similar to the reduction in conditioned HR responses seen after area 25 inactivation in the marmoset [38].

In summary, non-human primate area 25 manipulations can clearly modulate cardiovascular function during both neutral and stressful conditions, particularly within the parasympathetic domain. IL manipulation in rodents also modulates cardiovascular function indicating some degree of functional conservation across species, but the direction and consistency of these cardiovascular alterations differ, depending upon the nature of the manipulation and the type of stressor, making it difficult to compare directly with primates. Further investigations that control for these variables are required to fully compare the autonomic influences of rodent IL function with that of primate area 25.

### 2.2. Area 25 and Endocrine Function

As well as the autonomic component of visceral control, there is strong evidence that area 25, and associated prefrontal regions, also regulates the endocrine component via interactions with the hypothalamic–pituitary–adrenal (HPA) axis, the body’s primary stress response system. Unfortunately, as discussed below, surprisingly few studies have examined the neural correlates of HPA axis regulation in humans. Those that have, identify area 25 as an important contributor because it not only shows sensitivity to circulating cortisol levels but appears to be able to directly regulate HPA axis function.

Exogenous cortisol administration directly modulates the response of area 25 to sad picture stimuli, blunting sadness-induced activation [36]. This indicates that area 25 is sensitive to circulating cortisol. Furthermore, in young adolescents, salivary cortisol measurements during social stress positively correlate with elevated functional connectivity between area 25 and the salience network, including the dACC and bilateral anterior insula [53]. However, it is unclear whether this correlation specifically reflects the actions of cortisol on activity within this network, or the effects of the stressor per se or the effects of area 25 on HPA axis function. Stronger negative functional connectivity has also been observed between a region of the vmPFC (encompassing area 25, subcallosal area 24 and perigenual area 32) and the amygdala that was associated with higher cortisol levels [54]. It was proposed that this negative functional connectivity reflects the top-down regulation of the amygdala during stress, which subsequently modulates HPA axis activity. Conversely, however, the correlation could reflect the impact of elevated cortisol on network activity. Nevertheless, together, these findings highlight the complex interactions between prefrontal (including area 25) top-down regulation of the HPA axis, and its regulation by cortisol.

This complex relationship between area 25, the HPA axis and sensitivity to cortisol is also seen in non-human primates. High densities of glucocorticoid and mineralocorticoid receptors, which are sensitive to cortisol and other stress hormones, have been found in the vmPFC (including area 25) and lateral PFC of squirrel monkeys [55], indicating that these areas are sensitive to cortisol. The macaque also shows direct projections to central autonomic centers such as the hypothalamus, but also indirect projections to the adrenal medulla itself, through which it can regulate HPA axis activity. Recent anatomical tracing studies have used injections of rabies virus into the adrenal medulla and a survival time series analysis method to identify the third and fourth order neurons from areas 24c, 25 and 32 as the densest projections to the adrenal medulla. These regions are broadly similar to regions identified in human functional imaging studies related to autonomic modulation, negative affect and cognitive control, indicating that this medial region may mediate the effects of chronic stress on visceral function [56]. Consistent with this, metabolic activity within area 25 has been related to individual differences in HPA axis regulation in macaque monkeys [37]. Having been exposed to four situations of increasing stress for 30 minutes (home with cage-mate, home alone, human intruder exposure or foreign cage alone), macaques then underwent femoral venipuncture for cortisol levels together with - 2-deoxy-2-[^18^F]fluoro-Dglucose (^18^F-FDG) Positron Emission tomography (PET) scan. Area 25 was the only brain region in which activity correlated with cortisol output across different contexts. However, as already described for humans, the directionality of the relationship between area 25 activity and circulating cortisol remains unclear. This is because a positive relationship could reflect both stimulatory and inhibitory (negative feedback) processes co-occurring within the HPA axis. For example, area 25 activity could be correlated with cortisol output if it were providing a direct stimulatory input to the HPA axis, or if it were activated by increasing concentrations of circulating cortisol to exert negative feedback.

As in non-human primates, high densities of glucocorticoid receptors are found in the rodent vmPFC, and importantly, the directionality of the relationship between IL activity and cortisol levels has been identified in rodents, as IL manipulations have been shown to alter stress hormone activity in response to a stressor. Thus, after restraint stress, radiofrequency ablation of caudal IL increases adrenocorticotropic hormone (ACTH/corticosterone) levels, while corticosterone implants into IL reduce ACTH/corticosterone levels [57]. Neither manipulation had effects on baseline levels. Clearly therefore, the IL is acting to regulate the HPA axis. It should be noted that combined manipulations of IL and PL have also been shown to modulate corticosterone responses to some stressors, albeit in a different manner to IL alone, but these results are difficult to interpret as the precise area responsible for the effect is not known [58,59]. There is also evidence that glucocorticoids acting directly on IL can regulate the behavioral correlates of acute and chronic stress. Thus, a selective knockdown of glucocorticoid receptors within IL (not PL) increases immobility time in the forced swim test, a commonly used assay of depression-like behavior [40] (Figure 2Dii). However, it remains to be seen if administration of cortisol also directly alters IL activity, as seen in humans. 

### 2.3. Area 25 and Immune Function

In addition to the role that area 25 plays in the release and regulation of physiological factors such as stress hormones, emerging evidence also implicates area 25 as a key player in the orchestrated responses to immune challenges. In humans, elevated activity within area 25 and subcallosal area 24 has been associated with increased levels of interleukin-1β during grief elicitation [60]. Injection of the typhoid vaccine increases interleukin-6 levels and negative mood compared to placebo, and inflammation-associated mood-deterioration directly correlates with elevated activity in area 25, subcallosal area 24 and pgACC area 32 [61]. Depressed patients also show significantly increased numbers of microglial cells in area 25, suggestive of increased inflammation within area 25 itself [62]. However, further work is necessary—particularly in preclinical rodent and non-human primate models—to elucidate whether there is a clinically significant interplay between area 25 and the immune system. 

### 2.4. Summary

To conclude, area 25 clearly contributes to the regulation of the cardiovascular, endocrine and immune components of a co-ordinated visceral response, and as such, holds a vital integrative role across humans, non-human primates and rodents. Despite this, this visceral integration appears to be unconnected to the level of the intrinsic resting connectivity network related to internal processing in the brain. Thus, in healthy subjects, area 25 and the caudal scACC are generally not included within the default mode network; an intrinsic, distributed network of brain regions, including the posterior cingulate and rostral vmPFC, which shows correlated activity at rest, together with correlated task-dependent modulations [63,64,65] (but see [65,66,67] which do suggest area 25 involvement). However, it should be noted that area 25 can be preferentially recruited into the default mode network in depressed states. In these situations, the integrative visceral roles of area 25 could assume undue prominence [68] manifesting as suppression of parasympathetic outflow, elevated baseline HR and reduced baseline heart rate variability [29,69]. This is consistent with the preferential modulation of cardiovascular parasympathetic regulation after area 25 manipulations in humans and non-human primates, the evidence for autonomic dysregulation in depression, and the preferential effects of the novel antidepressant ketamine on the scACC, including area 25 [38,65,70,71]. Area 25 may therefore be a key node in the integration of negative mood and abnormal visceral regulation, a premise supported by two recent meta-analyses of neuroimaging data that have associated area 25 activity with functions attributed to the default mode network, including emotion processing, attribution of affective meaning and autonomic function, as well as mentalization and autobiographical memory [19,72,73,74]. If so, area 25 is in a unique position to subconsciously link bioregulatory states with their mnemonic and emotional mood states. 

## 3. Emotional Function and the Subcallosal Zone

### 3.1. Human Area 25 and Its Association with Negative Emotion and Anhedonia

#### 3.1.1. Non-Pathological Mood States

The subcallosal region of the ACC, including area 25, has received significant attention in the context of negative mood and depression. This is due to the high frequency at which functional and morphological changes within this region have been identified in studies of negative affect, and the function of this region as a point of integration between visceral, attentional and affective information important for homeostasis and allostasis. 

Suggestions that elevated area 25 activity may be relevant to disorders of enhanced negative emotion, in part, derive from studies implicating subregions of the scACC in transient states of sadness induced in healthy control subjects. In a comprehensive meta-analysis of earlier work that included 55 PET and fMRI emotion-induction studies across both positive and negative valence, induction of sadness was significantly associated with activation/increases in rCBF of an scACC region which partly encompassed area 25 [75]. Two important issues should be highlighted. First, only 46% of studies using sadness induction paradigms reported increased activity within the subcallosal region. This may be because of differences in the sadness provocation methods used. Earlier studies scanned participants during active generation of the sad state and yielded variable subcallosal activation [76,77,78]. In contrast, studies which scanned participants once the sad state was attained reported more consistent subcallosal activation [79,80]. Second, when subcallosal activation was reported to include area 25, closer inspection revealed activity to be focused in a more rostral area encompassing subcallosal area 24 rather than area 25.

Nevertheless, studies subsequent to this meta-analysis have identified elevated activity associated with negative affect in area 25. For example, increased activity within a region bordering areas 10 m, 32 m and 25 m positively correlated with an aggregate self-report score of individuals’ experience of negative affect over the previous month [81]. Moreover, assessment of the neural responses to sad pictures in healthy elderly individuals revealed elevated activity within the subcallosal region, extending along the rostro-caudal extent to include area 25, subcallosal area 24 and area 14 [82]. Of particular interest, is a recent study [36] in which area 25 was not only selectively activated in participants viewing sad stimuli, but this activity was reduced by hydrocortisone, highlighting the sensitivity of this region to circulating cortisol.

In addition to the use of pictorial or autobiographical stimuli to induce negative mood states, several studies have explored regional metabolism in the context of affective verbal processing. In these studies, regions of the medial PFC are robustly engaged by emotional words, irrespective of valence. However, these regions are typically more rostral than area 25, corresponding to the area 25/24 border zone, subcallosal area 24 and area 32 [83,84]. Tryptophan depletion has also been used to induce negative mood states in healthy controls [85]. Tryptophan is the precursor of serotonin (5-hydroxytryptamine; 5-HT), and rapid depletion of tryptophan reduces brain 5-HT. The effects of tryptophan depletion span cognitive and affective domains, although the magnitude of the affective change is variable, with some studies showing that healthy controls—even if given selective serotonin reuptake inhibitor (SSRI) medication—do not show any mood changes [86]. In one such study, acute tryptophan depletion increased rCBF bilaterally in area 25 that correlated with reduced mood, and simultaneously decreased rCBF in dACC/area 24 in a mood-independent manner [87].

An additional cluster of studies have implicated scACC in negative affect derived from social exclusion and rejection in adolescents and young adults. In these studies, social exclusion is typically induced using a virtual game termed Cyberball [88], in which participants are excluded from a ball-tossing game by other pre-programmed virtual participants. However, a meta-analysis of fMRI-measured brain activations during social exclusion, predominantly using the Cyberball task, identified positive correlations between social exclusion and activity centered on subcallosal area 24, rather than area 25 [89]. 

In summary, the location of activation within the scACC during negative mood states, including sadness, is quite variable and only sometimes is area 25 included. Indeed, a recent study used the cytoarchitectonic and chemoarchitectonic profile of ten human post-mortem brains to construct continuous and maximum probability maps of the distinct subcallosal fields for areas 25, 24, 32 and 33. Using a forward inference approach, the functional connectivity profile of each area was assessed and it was the subcallosal portion of area 24, rather than area 25, that showed consistent activation in psychological processes encompassing ‘sadness’ [18].

#### 3.1.2. Pathological Mood States

In one of the earliest studies to report a relationship between reduced volume of the scACC in both unipolar and bipolar depressed subjects [90], the region of interest lay relatively rostral, at the border between subcallosal area 24 and perigenual area 32. Indeed, in an extension of this initial study, the region of interest still lay anterior to area 25 [91]. A subsequent study measured the volumes of both an ‘anterior’ region corresponding to subcallosal area 24, and a ‘posterior’ region corresponding to area 25 [92]. Here, the volume of left area 25 was smallest for patients with psychotic major depression compared to schizophrenics and healthy controls, whereas volumes of the subcallosal area 24 did not differ between groups. More recently, several studies have identified reductions in subcallosal volume encompassing varying portions of area 24 [93], area 25 [94], or both [95,96,97].

Besides reports of volume change, there have also been reports of functional abnormalities. In early studies, hypoperfusion in broad regions encompassing the scACC was consistently reported in Single Photon Emission Computed Tomography (SPECT) and PET studies [98,99,100,101,102]. However, these studies are in part confounded by the influence of volumetric changes within area 25 of patients suffering from Major Depressive Disorder [92]. When corrected for regional atrophy, area 25 does not show reduced activity—instead, rCBF measurements suggest activity is normal [80] or elevated [103]—although in the latter study the subjects were treatment resistant and the elevated activity appeared more rostral than area 25. However, more consistent are the reductions seen in area 25 activity in treatment responders following deep brain stimulation [103], fluoxetine [80], and the placebo effect [104].

Primary increases within subregions of the scACC in depressed populations have also been reported in functional studies. For example, when comparing patients with Major Depressive Disorder to non-depressed individuals, greater activity within area 25 was associated with the processing of sad faces, whereas greater activity within subcallosal area 24 was associated with processing of happy faces [105]. A subsequent meta-analysis of resting-state and emotion-activation studies found increased activity within right area 25 of depressed patients when exposed to positive emotional stimuli [106], and decreased activity following SSRI treatment. However, a more recent meta-analysis found decreases in area 25 associated with emotional processing tasks in depressed patients, although this study did not separate the positive and negative domains of emotion processing [107].

Besides negative mood, a key symptom of depression is anhedonia, the loss of interest and pleasure in all or almost all activities. It is highly prevalent in depression [108] and is a negative prognostic indicator [109,110], often associated with treatment resistant depression. However, area 25 is seldom identified in anhedonia-related neuroimaging studies. Functional activity changes associated with anhedonia are typically more rostral than area 25, encompassing regions such as area 10/24 and perigenual area 32 [111]. A recent study did however investigate abnormal connectivity patterns in a more posterior subcallosal region associated with altered activity in mood and anxiety disorders based, on an activation-likelihood estimation meta-analysis. The connectivity of the ROI with key reward-related regions (including the nucleus accumbens and ventral tegmental area [VTA]) was negatively correlated with anhedonia during pleasant music listening, but not anxiety levels, whereas resting state activity changes within the ROI did not differentiate between the two symptom clusters [112]. However, this ROI mainly included area 24 and was still rostral to area 25. 

#### 3.1.3. Neurobiological Models of Depression with a Focus on Human Area 25

There are several influential neurobiological models of depression that directly implicate vmPFC dysfunction in its etiology and/or pathogenesis, namely the limbic-cortical, cortico-striato-pallido-thalamic (CSPT) and default mode network models. These models are not mutually exclusive and are overlapping in terms of the neurobiological substrates being implicated, and the consequences that dysfunction within these structures has on behavior, physiology and cognition. We will focus on the role of area 25 within these models. 

*The limbic-cortical model* was formulated in order to link impairments in cognition to sustained alterations in mood states characteristic of depression [113]. It focused on hypoactivity in a dorsal compartment proposed to be principally involved with the attentional and cognitive features of depression, including dm/dlPFC, area 24, parietal cortex and the dorsal striatum. Hyperactivity in a ventral compartment, consisting of limbic and paralimbic structures including area 25, was proposed to mediate the vegetative and somatic aspects of depression. Finally, the rostral cingulate, corresponding to perigenual areas 24 and 32, [113] was proposed to regulate the interaction between the dorsal and ventral compartments. Depression was then hypothesized to result from a failure of the coordinated interactions within and between compartments. 

One of the most promising treatment modalities developed from this model is deep brain stimulation. In 2005, it was reported that deep brain stimulation targeting area 25 ameliorated symptoms of depression in four out of six individuals with treatment refractory depression [103]. Although an industry-sponsored trial utilizing deep brain stimulation of area 25 has failed in recent years [114], this has not stalled further investigation, with subsequent work refining neurosurgical targeting techniques and identifying potential biomarkers which might predict treatment response. Tractography imaging techniques to identify similarities in electrode contacts within deep brain stimulation responders have also highlighted the importance of four white matter bundles underlying area 25 [115]. This approach is proving valuable in identifying optimal deep brain stimulation targets to achieve antidepressant responses [116].

*The cortico-striato-pallido-thalamic model* posits abnormal activity in the CSPT circuitry to explain, at least in part, the clinical symptoms and cognitive deficits associated with depression. CSPT loops connect regions of the PFC with the basal ganglia and thalamus in a parallel but overlapping manner to support a multitude of behavioral and cognitive functions [117]. Evidence for the importance of CSPT circuitry in mood disorders includes: (i) structural and functional imaging studies that show evidence of alterations in CSPT components associated with depression [118,119,120]; and (ii) a higher prevalence of depression associated with neurodegenerative and vascular diseases that involve CSPT circuitry [121,122,123]. The ventral caudate and nucleus accumbens (forming, together with the olfactory tubercle, the ventral striatum) are arguably the most consistently implicated striatal subregions in depression. Patients with remitted depression show hyperactivation of the caudate and accumbens during negative picture viewing [124], and currently depressed patients show hypoactivation of the accumbens and ventral caudate during rewards [125,126]. Aberrant ventral striatal functional connectivity also predicts future risk for developing depression [127].

Given the anatomical evidence that area 25 and adjacent vmPFC projects strongly to the ventral striatum [117], area 25-ventral striatal limbic circuitry has been explored in the context of CSPT changes associated with depression. Meta-analytic approaches have consistently identified volumetric abnormalities within these limbic CSPT circuits: reduced volume in the PFC—especially area 25 and OFC—together with reduced volume in the ventral caudate and putamen [128,129]. However, a meta-analysis of functional resting-state network connectivity in depression identified reduced connectivity between subcallosal activity rostral to area 25 and the ventral striatum [130].

Finally, in the *default mode network model*, increases in functional connectivity between the caudal vmPFC, specifically area 25 and nodes (rostral vmPFC and posterior cingulate cortex) within the default mode network, have been reported in people with depression [131]. Thalamic involvement is also evident, with increased connectivity between area 25, the mediodorsal (MD) thalamus and the default mode network, which has also been linked to higher levels of rumination [132,133,134]. These findings have led to the proposal that increased functional connectivity between this network (involved in biasing towards self-referential thinking processes) and area 25 (supporting negatively affectively-laden behavioral withdrawal) result in pathological rumination: self-focused, negatively valenced and withdrawn thinking processes [132]. However, since area 25 does not directly project to nodes of the network [135], but does project to the MD thalamus (which itself projects to the network nodes) it is suggested that the increased correlation of activity between area 25 and the default mode network is mediated by projections through the MD thalamus.

Altogether, whilst there is considerable correlative evidence for a role of area 25 in negative mood states in humans, direct evidence is limited. Whilst there have been a number of important studies describing the behavioral effects of varying levels of damage to human vmPFC (for review see [136], the selective contributions of area 25 could not be determined. Perhaps surprisingly, few non-human primate studies have addressed the contributions of area 25 to emotion and its regulation and thus, until recently, the majority of our understanding had come from studies of the IL in rodents. The findings from monkeys and rodents will now be described and their translatability to one another, and to human studies, will be discussed.

### 3.2. Monkey Area 25 and Its Association with Negative Emotion and Anhedonia

#### 3.2.1. Neurophysiological Correlates of Reward and Punishment

One of the earliest studies in macaques to record in mid to caudal regions of area 25 found that the neurons had very low spontaneous firing rates, and failed to respond to tastes and olfactory cues, or to reward associated stimuli on a visual discrimination reversal task, including faces. Of the 93 recorded neurons, 11 showed an increase in responding from zero to approximately four spikes/sec during slow wave sleep [45]. Consistent with the lack of responsiveness to appetitive cues, a more recent study also showed that area 25 neurons displayed little response to appetitive conditioned stimuli (CSs; i.e., visual cues paired with reward) and unconditioned stimuli (USs; i.e., reward) during appetitive blocks of a Pavlovian task [137]. However, they did signal both aversive CSs and USs, in the form of visual cues paired with an air puff to the face. In contrast, more ventrally located neurons [1,2] were persistently more active in appetitive blocks. When recordings were made more rostrally in perigenual regions of area 25, neurons responded to a wide range of variables during a gambling task, both positive and negative, although there was a bias towards encoding of negative outcomes [138]. An overlapping region of recordings in this perigenual zone identified neurons as being more sensitive to internal factors, such as satiation, rather than external factors, such as visual cues [139]. Whether these differences across studies reflect changes in function between rostral and caudal and dorsal and ventral sectors of area 25 remain to be determined.

#### 3.2.2. Area 25 Manipulations and Threat

**Figure 3:**
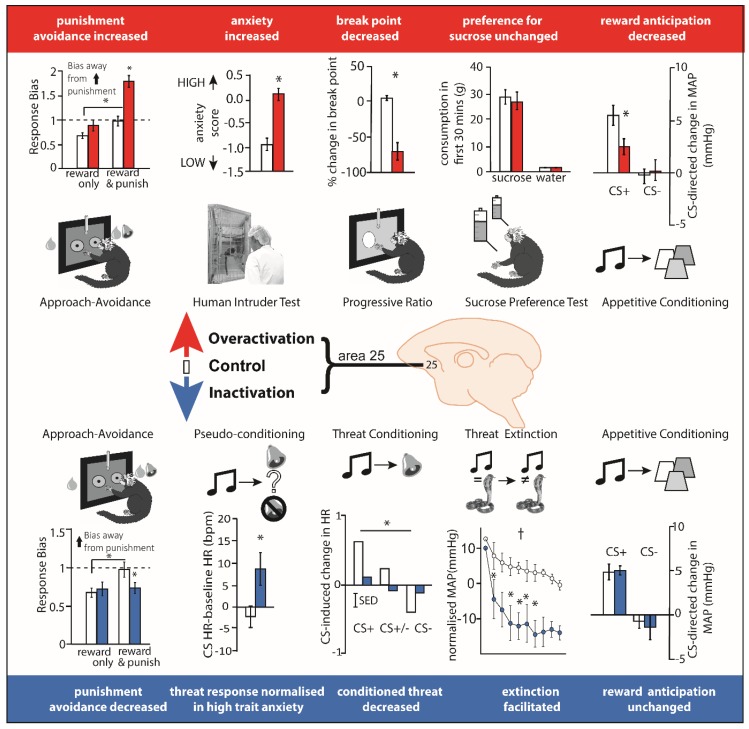
Over-activation and inactivation of area 25 in marmoset has largely opposing effects on tests measuring the regulation of responsivity to rewarding and punishing stimuli. Top from left. Over-activation via presynaptic glutamatergic disinhibition increased the bias away from punishment in an approach-avoidance decision making paradigm [140], and over-activation via inhibition of the excitatory glutamate amino acid transporter (EAAT2; with dihydrokainic acid [DHK]) increased anxiety-like behavior in the human intruder paradigm, increased the break point in a progressive ratio task, left sucrose consumption unaltered, and blunted the anticipation of food reward during appetitive conditioning [141]. Bottom from left. In contrast, inactivation with GABA A and B agonists (muscimol and baclofen) decreased the avoidance of punishment during approach-avoidance decision making [140] and reversed the blunted cardiovascular responsivity to threat in high trait anxious animals [46]. Inactivation also blunted the threat-induced anticipatory increases in heart rate and vigilant scanning (not shown) during Pavlovian threat conditioning and enhanced the rate of threat extinction [38], but had no effect on reward anticipation during appetitive conditioning [141]. *, *p* < 0.05, ^†^, *p* < 0.05, main effect of manipulation; error bars indicate SEM. Thus, in general, over-activation blunted appetitive responses whilst enhancing threat-induced responses whilst inactivation primarily dampened threat-induced responses.

Recently, pharmacological studies have been carried out in marmoset monkeys that temporarily inactivate or activate area 25 (see Figure 3). Inactivation with GABA A and B agonists (muscimol and baclofen) (i) reduced the expression of behavioral (orienting/scanning) and cardiovascular (blood pressure and heart rate) conditioned threat responses during Pavlovian discriminative conditioning when a previously neutral stimulus (i.e., an auditory cue) became associated with an aversive event (i.e., a loud noise and (ii) accelerated extinction of conditioned cardiovascular and behavioral responses, when the conditioned stimulus no longer predicted the aversive event (i.e., rubber snake) [38]. These findings suggest that non-human primate area 25 normally acts to drive Pavlovian cardiovascular and behavioral responses during threatening situations. 

The directionality of the effects of area 25 manipulations were conserved in the instrumental domain too. Using a touchscreen approach-avoidance decision-making task where marmosets responded for rewards with the potential for punishment, inactivation of marmoset area 25 reduced punishment avoidance. Conversely, enhancing pre-synaptic glutamate release within area 25 (using a combination of mGlu_2/3_ and GABA_B_ receptor antagonists) enhanced punishment avoidance [140]. The increased sensitivity to punishment seen when glutamate release in area 25 was enhanced, causally relates elevated activity in area 25 to negative decision-making biases observed in individuals with depression [142,143]. Consistent with these findings, increasing activity within marmoset area 25 using an alternative method, namely inhibiting the excitatory amino acid transporter-2 (EAAT2) to reduce glutamate reuptake (using dihydrokainic acid [DHK],) enhanced marmoset responsivity on the human intruder test, a classic method of measuring anxiety-like behavior in a primate which assesses the behavioral responses to an unfamiliar human that elicits uncertainty [141]. Taken together, these data suggest that inhibiting activity within marmoset area 25 reduces the behavioral and cardiovascular correlates of negative affect, whereas increasing activity has the opposite effect, and enhances these correlates. These effects may reflect the behavioral and physiological output of the neural bias in encoding negative outcomes described in electrophysiological studies. 

#### 3.2.3. Area 25 Manipulations and Reward

The only lesion study to target area 25 in a non-human primate implicated this region in the maintenance of arousal in anticipation of positive rewarding outcomes. Ablation of area 25 in macaques impaired their ability to sustain autonomic (pupillary dilation) arousal during a trace interval between an appetitive CS and US [144]. Disruption of this function could be relevant to the reduced reward processing associated with depression, broadly referred to as anhedonia (involving a reduced ability to experience pleasure, together with reduced anticipation and motivation). However, as discussed above, reduced activity in this area is more consistently associated with recovery from depression. Moreover, given that this study in macaques used ablations, it is possible that the effects reported were a consequence of damage to fibers of passage, especially since this region is a major conduit for a number of fiber bundles carrying fibers to and from the cortex [115].

More recently, temporary pharmacological manipulations in area 25 of marmoset monkeys that selectively target neurons intrinsic to area 25, without affecting fibers of passage, has revealed that over-activation of area 25 not only enhances negative reactivity but also blunts anticipatory and motivational appetitive arousal (see Figure 3). The measurement of cardiovascular and behavioral responses during the CS ‘anticipatory’ and US ‘consummatory’ period of a Pavlovian appetitive conditioning task revealed selective blunting during the anticipatory period only following DHK-induced increases in glutamate release in area 25. The same infusions also caused an earlier breakpoint on an instrumental progressive ratio schedule of reinforcement [141] reflecting a reduction in the willingness to work for reward. The finding that inactivation of area 25 (using the GABA A and B agonists muscimol and baclofen) had no effect on anticipation suggests that area 25 activity is not necessary for reward-related anticipatory arousal but, when activated, has an inhibitory effect. An obvious question arising from these findings is what factor(s) naturally cause(s) activation of area 25? Given the relationship between area 25 and the HPA axis, described above in ‘Area 25 and endocrine function’, stress may be a key factor. 

^18^F-FDG PET imaging has provided insight into the changes in downstream brain regions caused by area 25 over-activation. These include increases in metabolic activity in the dorsomedial PFC and insula, but decreases in activity within a region encompassing the nucleus of the solitary tract and brainstem 5-HT neurons [141], all regions implicated in the networks of depression [132]. Of particular relevance to our understanding of current treatments of depression, peripheral ketamine, a recently discovered glutamate based anti-depressant with particular efficacy in treating reward-related deficits [145,146,147], ameliorated the anhedonia-like symptoms induced by area 25 over-activation. Not only did ketamine restore cardiovascular and behavioral anticipatory arousal but it also reversed the changes in the network. Thus, over-activation of area 25 produces both anxiety-like and anhedonia-like behavioral symptoms in a monkey and, consistent with clinical reductions of area 25 activity in treatment responders, ketamine ameliorates the anhedonia-like effects. 

#### 3.2.4. Area 25 and Its Interactions with the Anterior Hippocampus

Pharmacological intervention studies in marmosets have also probed the importance of area 25 in the wider network of structures important for regulating affective behavior. Initial studies have focused on the anterior hippocampus (aHipp), given the importance of connectivity between these regions in psychiatric disorders [148]. In one study, aHipp activations in high trait anxious marmosets were shown to reduce the marmosets’ anxiety-like behavior to uncertain threat in the form of a human intruder, as well as to normalize their blunted behavioral and cardiovascular response to unpredictable aversive loud noise. Simultaneous inactivation of area 25, but not area 32, however, blocked these anxiolytic effects, areas which, when inactivated independently, reduced or had no effect on anxiety, respectively [46]. Simultaneous inactivations of area 25 have also been shown to block the ability of aHipp activations to reduce punishment avoidance on an approach-avoidance instrumental decision-making paradigm, despite area 25 reducing punishment avoidance when inactivated independently [140]. In both examples, activation of aHipp and inactivation of area 25 independently reduced threat-induced responses, but when occurring simultaneously these anxiolytic effects were abolished, thereby highlighting the importance of the interaction between these two regions in the regulation of negative emotion. However, how their effects are orchestrated within the wider network of structures known to be involved in regulating responsivity to threat is a key question for future studies. For example, the amygdala, striatum, OFC and ventrolateral prefrontal cortex (vlPFC) have all been implicated in approach-avoidance decision making [149,150,151,152]. Indeed, using the exact same paradigm, interactions between the OFC and amygdala in marmosets have been shown to contribute to the long-lasting mnemonic effects of punishment on decision making, without influencing decision making at the time of punishment per se [149]. Different again are the effects of inactivation of the vlPFC, which acts to enhance avoidance of punishment on decision making at the time of punishment. Since the aHipp projects to the vlPFC as well as area 25 [14] and there is weak to moderate connectivity between area 25 and vlPFC [1], investigating the nature of the interactions between these structures on approach-avoidance decision making and other threatening contexts is an important next step.

### 3.3. Rodent Infralimbic Cortex and Its Association with Negative Emotion and Anhedonia

#### 3.3.1. Conditioned Threat and Its Extinction

Experimental studies in rodents have implicated IL in a range of different behavioral functions, but some of the most extensive investigations have focused upon its role in the extinction of conditioned threat. Early experiments in rodents assessed the effects of broad lesions to mPFC (including PL, IL, Medial OFC and ACC) on the extinction of threat memories, as measured by the low level of conditioned freezing displayed to a CS that was no longer paired with foot shock, and found these lesions severely impaired extinction, without an effect on the acquisition of conditioned freezing [153]. Subsequently, lesions restricted to the IL confirmed this region’s role in the successful recall of extinction while, lesions that spared most of the IL did not have an effect, suggesting that the IL is the critical mPFC sector necessary for recalling extinction memories [154]. Since this lesion work, electrophysiological, microstimulation and pharmacological inactivation studies have also probed the specific contributions of the IL and PL to threat regulation. In seminal work, reviewed in Milad and Quirk [155], recordings from IL neurons during acquisition, extinction and extinction recall phases revealed that IL neurons fired only when recalling a CS/noUS association on extinction recall days. The degree of firing correlated with successful recall of this association: the more IL neurons fired, the less rodents froze. Pharmacological inactivation studies have since extended this work by causally implicating IL in extinction and extinction recall and indicate that IL is a key player in the inhibitory mechanisms which may ‘gate’ information flow within downstream structures—such as the amygdala—during CS-noUS learning and retention [156,157]. These effects are not just restricted to conditioned freezing, as IL inactivation also disrupts extinction recall when avoidance is the conditioned response [158] and conversely, IL activation during extinction using d-cycloserine can facilitate re-extinction of the conditioned freezing response the next day [159].

**Figure 4:**
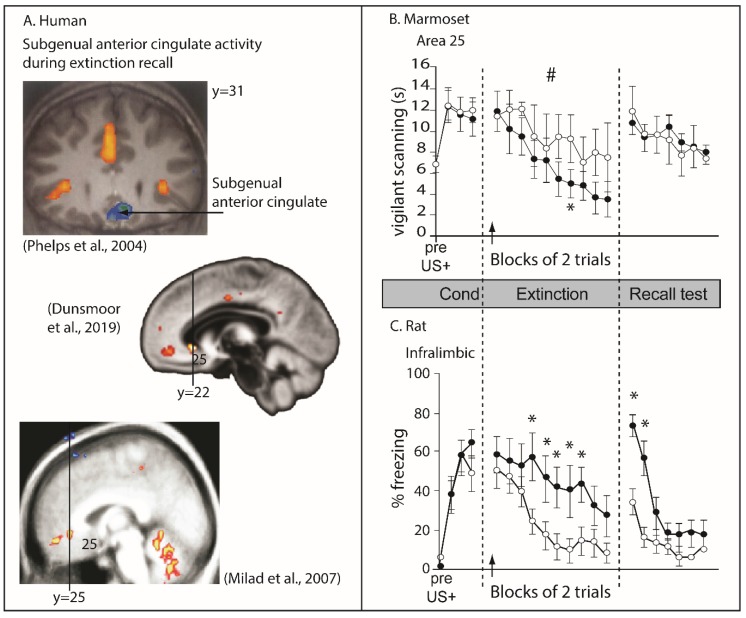
Re-thinking the role of area 25 in threat extinction in humans, marmosets and rats. (**A**) Human neuroimaging of extinction recall has identified regions of the subcallosal zone (scACC) in which the deactivation induced by the conditioned stimulus, CS+ is blocked following successful extinction recall [160,161,162]. However, these regions of activity are generally more rostral than area 25. (**B**) In marmosets, inactivation of area 25 with GABA A and B agonists (muscimol and baclofen; closed circles) hastened the behavioral extinction of an aversive (rubber snake) Pavlovian conditioned association [38]. In contrast (**C**) IL inactivation in rats (muscimol; closed circles) impeded the behavioral extinction and extinction recall of conditioned footshock. Redrawn from Sierra-Mercado et al., [157]. *, *p* < 0.05; #, *p* < 0.05, manipulation × CS interaction; error bars indicate SEM. Arrow indicates point of inactivation.

Although neuroimaging studies in humans have been interpreted to support the role of this region in recall of extinction of conditioned threat responses [160,161,162], as measured by skin conductance responses, the regions of altered activity are far more rostral than area 25, in one case almost at the level of the genu of the corpus callosum. Thus, although a region within vmPFC in humans shows correlated activity with extinction recall, it does not correspond to area 25 (Figure 4A). Moreover, as described above, inactivation of area 25 in marmosets facilitates extinction rather than impairs it, and in contrast to the impaired extinction recall in rodents, has no apparent impact on its recall (compare Figure 4B and Figure 4C). Incidentally, area 32 inactivation in marmosets also produces opposite effects to those reported following inactivation of the putative functionally homologous region in rats, namely, the PL, with area 32 inactivation retarding extinction in marmosets as opposed to the impaired threat recall after PL inactivation in rats. There are at least two explanations for this discrepancy. The first is that the IL in rodents is not functionally homologous to primate area 25. The second is that the task design used in marmosets to study extinction of conditioned threat, although developed to match that of the rodents as close as possible, was not identical and performance relied on different psychological processes to those of the rat, that were differentially sensitive to area 25 inactivation. If the latter, then at the very least these results call into question the hypothesis that area 25 is essential for threat extinction and in particular threat extinction recall. Instead, it suggests that the underlying function of this region, when dysregulated, can have mixed effects on the extinction of conditioned threat responses, e.g., facilitative or antagonistic, presumably depending upon the precise context in which the conditioned threat is learned and modulated. Before considering, however, what the underlying function of IL might be, the contribution of IL to additional behavioral domains will be discussed.

#### 3.3.2. Depression-Like and Anxiety-Like Symptoms

Besides extinction of conditioned threat, altered activity in rodent IL has been implicated in putative depression-like (despair) and anxiety-like symptoms, as measured across a range of paradigms including forced swim, tail suspension (despair-like tests) and novelty suppressed feeding, elevated plus maze and open field (anxiety-like tests). Here, however, the findings have been contradictory. An early report [163] revealed anti-depressant effects of inactivation of IL induced by the GABA agonist, muscimol, on the forced swim test in normal rats and those bred for high anxiety; activation of this area by the GABA antagonist, bicuculline, had no effect. In contrast, more recently, activation of IL induced by DHK [164] produced anti-depressant and anti-anxiety-like effects on the forced swim test and novelty suppressed feeding tests, respectively. A similar effect is seen following an acute optogenetically-induced activation of the pathway between IL and the amygdala [165], or following sustained optogenetically-induced activation of area 25 globally [166]; in which case the anti-anxiety effect is long lasting, being seen 24 h later and beyond. Different again, however, activation of IL induced by a GABA-A antagonist, induced anxiety-like behaviors on elevated plus maze, open field and novelty-suppressed feeding [167], as did an acute optogenetic activation of IL pyramidal neurons [168]. Acute versus more sustained activation of IL may contribute to the variation in the results, with acute effects tending to be anxiogenic whilst prolonged effects are more likely anxiolytic. However, very recently, sustained activation induced by genetic knockdown of the astrocytic glutamate transporter GLAST/GLT-1 expression, induced a depressive-like phenotype on the tail suspension and forced swim tests [169]. Other contributory factors to the variation in findings may therefore include whether the animal was tested in its subjective ‘night’ or ‘day’, with the former being likely to enhance the level of stress experienced; however, this information is not always provided. Prior experience with other stressors may also impact on overall subjective experience. Finally, whether IL was targeted via cannulas passing through the PL or not has also been suggested to be an important consideration, since there may be infusion spread up the tract which can only be ruled out by direct comparison with infusions into the PL [170]. 

The contribution of IL to reward processing domains have also been investigated. Overall, inactivation of the IL tends to increase, and activation of the IL reduce, reward driven behaviors in a variety of contexts. These include the spontaneous recall and reinstatement of Pavlovian and instrumental appetitive responses following extinction (reviewed in [171]) that are increased by lesions/inactivation of IL, comparable to that seen following extinction of Pavlovian threat responses reviewed above. Conversely, activation with d-cycloserine induces the opposite effect [172]. In addition, activation with DHK increases the threshold for lever pressing for electrical brain stimulation and increases the latency to begin consuming sucrose [173], all effects consistent with putative symptoms of anhedonia and opposite to the anti-depressant effects on the forced swim test described above [164]. It should be noted however that in the latter [173], cannulas passed through the PL to reach the IL, but infusions were not compared with equivalent manipulations of the PL. Nevertheless, additional support of a pro-depressant effect of IL activation in the reward domain, sustained activation by knockdown of GLAST/GLT-1 expression reduced sucrose consumption, an effect that was not due to PL involvement [169]. Moreover, IL activation using the GABA antagonist bicuculline dampens the intense eating behavior generated by glutamate disruptions in the nucleus accumbens shell [174]. In contrast, acute inactivation of the IL does not affect the break point of a progressive ratio schedule [175] and excitotoxic lesions of the IL have no effect on acquisition of appetitive Pavlovian conditioned autoshaping [176]. Thus, in summary, as seen in marmosets, activation of IL very often has broader effects on rewarded behaviors than effects of inactivation and in general, activation tends to dampen reward driven responses. 

Without direct comparisons across laboratories using identical paradigms and pharmacological doses of drugs such as DHK and muscimol, or light stimulation parameters in the case of optogenetics, it is difficult to reconcile these mixed pro-depressant and anti-depressant findings on tests of learned helplessness and reward, and integrate them with the effects on conditioned threat extinction. Moreover, it is problematic to interpret changes in apparently ‘normal’ behavioral responses on a given test as anti-depressant, when there is no evidence that performance is ‘depressed-like’ in the first place. There are many reasons why an animal may spend more time swimming in a forced swim test and more time eating in the novelty suppressed feeding test other than they are displaying, respectively, a less ‘depression-like’ and ‘anxiety-like’ phenotype [177]. Future studies should determine whether IL activation shows similar effects in a variety of other contexts designed to measure depression-like and anxiety-like behaviors to determine the consistency of these effects e.g. anticipatory, motivational and decision-making contexts related to reward processing and cognitive and affective negative biases associated with anxiety. In addition, these studies should include the measurement of multiple indices that extend beyond behavior to include physiological measures such as cardiovascular reactivity and cortisol levels since, as described above, depressive and anxiety-like states have a marked impact on physiology. Moreover, if testing for anti-depressant-like effects, it is more informative if a depressive-like phenotype is induced first, such as that produced by learned helplessness models [178].

Given the marked variation between rodent studies with regards anti-depressant and anxiolytic behavior, it is difficult to compare results with those described in marmosets. Comparisons are really only informative when the same pharmacological manipulation is made across species. Thus, DHK (overactivation)-induced anhedonia-like effects on responding to brain stimulation in rats [173] appear consistent with the anhedonia-like effects of DHK infusion into area 25 on the instrumental progressive ratio test in marmosets. Apparently different though is the deficit in sucrose consumption reported in rats, but not in marmosets. However, it should be noted that sucrose consumption per se was intact in rats and only a DHK-induced increase in the latency to begin drinking was observed. The latter could be interpreted as an anticipatory effect, which would then be consistent with the DHK-induced effects on anticipatory responding in marmosets. Nevertheless, it is still difficult to reconcile the hypothesized anxiolytic/anti-depressant effects of rodent IL DHK in the novelty-suppressed feeding test [164], with the DHK-induced increase in anxiety-like responses to an uncertain threat in the human intruder test in marmosets [141]. Such discrepancies can only be resolved in future studies using more comparable tests and with detailed comparison of the effects of DHK on network activity across species.

#### 3.3.3. Stress and Its Controllability

Another emotion-related domain that involves both the IL and PL is the behavioral immunization effect of learned control over a stressor. Those animals that learn to run in a wheel to escape tail shock subsequently learn to avoid shock in other apparati (typically a shuttle box), in contrast to animals that learn that their attempts to escape a shock are futile, and then go on to fail to avoid subsequent shuttle box shocks [178]. This immunization effect produced by the experience of control over a stressor is seen across a range of contexts including aggression, social dominance, immobility, neophobia, threat conditioning and extinction [179]. PL appears most central to these effects since inactivation of PL disrupts immunization effects on both social and shuttle box behavior, whereas IL inactivation only blocks immunization effects on social behavior [180]. Enhanced release of serotonin in the dorsal raphe nucleus (DRN) and forebrain terminal regions is implicated in the learned helplessness effect [179], but only neurons in the PL (not IL) projecting to the DRN show selective activation to escapable stress [181]. Thus, the role of the IL in the behavioral immunization effect remains less clear. 

However, the sensitivity of the IL to stress per se does appear clear. Whilst chronic stress can induce morphological changes within the PL and IL [182], the IL appears particularly sensitive, with even acute stress causing apical dendritic retraction, reduced spine-induced learning and disrupted threat extinction [183,184]. Moreover, intermittent stress in early adolescence increases the serotoninergic innervation of IL, but not PL, and promotes the emergence of an anxious phenotype in adulthood, although whether these two outputs were related was not investigated [183]. 

#### 3.3.4. A More Complex Role for IL in Behavioral Control

Given the varied effects of experimental lesions or temporary inactivation of the IL and PL across the appetitive/aversive and cognitive/emotion domains, recent critiques have attempted to integrate these diverse findings [185,186] by taking into account the role of the IL in the ability of well-trained responses to dominate behavior. As summarized by Sharpe and Killcross [186], lesions of IL prevent appetitive instrumental responding becoming habit-like and insensitive to alterations in the valuation of the goal, following over-training [187], and inactivation re-instates goal directed sensitivity of over-trained instrumental responses [188]. They also prevent over-trained responses from disrupting the ability of contextual cues from an under-trained task to resolve response conflict in a rat version of the Stroop task [189]. Accordingly, Sharpe and Killcross [186] propose that a parsimonious explanation for IL function is that it acts to promote performance of well-trained responses that are context independent and reflect the animal’s long term experience with the current contingencies. This expands upon a previous synthesis that suggested that IL inhibited previously established goal directed actions [185]. While they acknowledge some caveats with their current account, nevertheless it makes the point that high-order cognitive functions within this region are likely to have variable effects on tests of threat and reward driven behaviors depending upon the range of cues, responses and contingencies that may be in operation. Certainly, a recent study inactivated, independently, neuronal ensembles related to either food seeking or the extinction of food seeking within rat vmPFC, (targeting the IL in particular) and revealed the opposing effects that such manipulations could have on food seeking behavior [171]. It remains to be determined if similar effects can be seen with respect to threat-driven behaviors in rodents.

## 4. Summary and Future Directions

**Figure 5:**
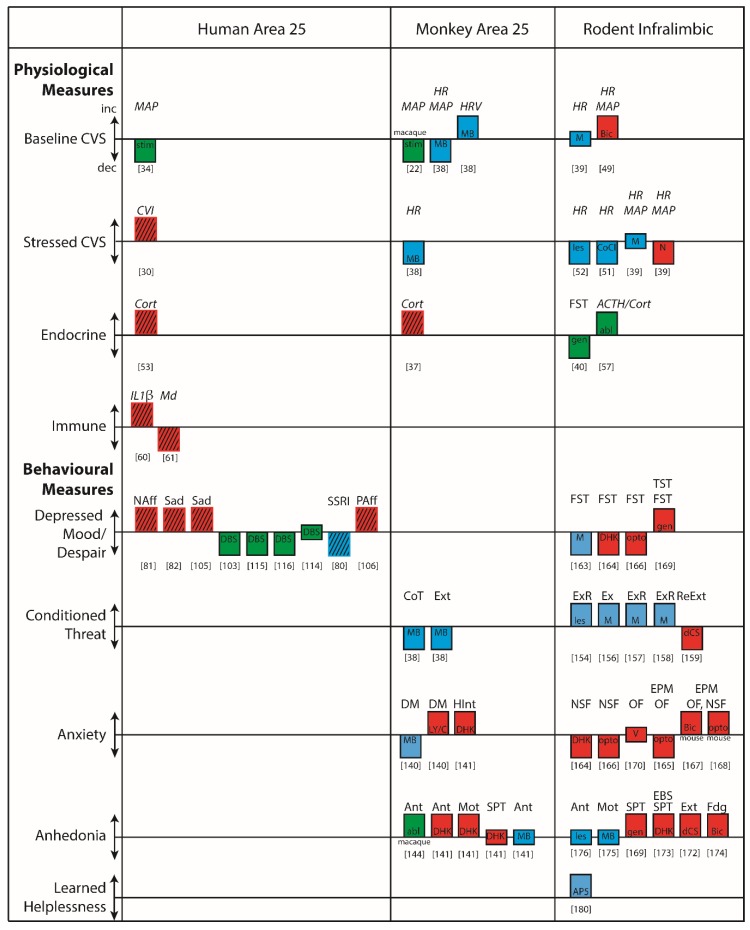
A summary of the physiological and behavioral functions associated with human and monkey area 25 and rodent infralimbic (IL). These representative studies illustrate, (i) the similarities and differences in the functional effects of manipulations of area 25 in monkeys and IL in rodents; (ii) how these map onto effects in humans; and (iii) where there are gaps in our knowledge. Blue bars denote reductions in activity, red bars denote increases in activity whilst green bars denote either that the direction of effects in area 25 are unclear, or that the effects may not be specific to area 25. Thus, if red and blue bars are going in the same direction (as in rodent despair), or if the same colored bars are going in opposite directions (as in rodent anxiety), the results are inconsistent. Headings above the bars indicate physiological measure if in italics, and the behavioral paradigm if non-italic. Hatching indicates correlations rather than manipulations. In terms of physiology, there is reasonable correspondence between monkey and rodent with respect to the reductions/increases in cardiovascular activity following reductions/increases in area 25/IL activity, especially during stress; although there are exceptions (see [39]). Any correspondence with the effects of stimulation in humans is unclear, however, because the excitatory versus inhibitory effects of stimulation on area 25 activity are unknown; if indeed the effects are due to changes in area 25 at all, since effects on fibers of passage cannot be ruled out. In relation to cortisol, there is agreement between correlatory findings in humans and monkeys that indicate positive correlations between cortisol levels and area 25 activity in monkeys and area 25 functional connectivity in humans. However, this similarity does not extend to rodents [57], as radiofrequency ablation of the IL increases corticosteroids; although whether the ablation effects are specific to the IL cannot be determined. Whilst immune function is related to area 25 activity in humans, this hasn’t yet been addressed in monkeys or rodents. With respect to behavior, changes in activity in area 25 in relation to depression can be variable, but successful treatment, especially in treatment resistant patients following DBS, is very often associated with reductions in area 25 activity. In line with this, the most consistent effects in both monkey area 25 and rodent IL are the overactivation-induced anhedonia-like effects. In contrast, the effects in monkeys and rodents of area 25/IL manipulations on conditioned threat responses and their extinction appear opposite, while in rodent studies the effects on despair-like and anxiety-like behaviors are inconsistent. Abl, ablation manipulation; AP5, AP-5 (NMDA antagonist); ACTH, adrenocorticotrophic hormone; Ant, Anticipatory arousal; Bic, Bicuculline (GABA_A_ antagonist); CoCl, Cobalt chloride (silences activity); CoT, conditioned threat; Cort, Corticosterone; CVI, Cardiac vagal index of heart rate variability; CVS, Cardiovascular system; DBS, Deep brain stimulation; dCS, d-Cycloserine (NMDA co-agonist); DHK, Dihydrokainic acid (EAAT2 inhibitor); DM, Decision making (approach-avoidance); EPM, Elevated plus maze; Ext, Extinction; ExR, Extinction recall; Fdg, Feeding behaviour; FST, Forced Swim Test; Gen, genetic manipulation; HInt, Human intruder test; HR, Heart rate; HRV, heart rate variability; IL1β, Interleukin 1β; Les, lesion manipulation; LY/C, LY341495 (mGluR2/3 antagonist) and CGP52432 (GABA_B_ antagonist); M, Muscimol (GABA_A_ agonist); MAP, mean arterial pressure; MB, Muscimol (GABA_A_ agonist) and baclofen (GABA_B_ agonist); Md, Mood; Mot, Motivational arousal; NAff, Negative affect; NSF, Novelty suppressed feeding, OF, Open Field test; Opto, Optogenetic manipulation; PAff, Positive affect; ReExt, Re-extinction; Sad, Response to sad faces; SPT, Sucrose preference test; SSRI, Selective serotonin reuptake inhibitor; Stim, Stimulation manipulation; TST, Tail suspension test; V, Veratrine (Sodium channel activator). Numbers indicate the relevant reference.

Fundamental to our understanding of area 25 in disease states is gaining an insight into the physiological, behavioral and cognitive functions requiring an ‘on-line’ area 25 (summarized in Figure 5). Encouragingly, the finding that area 25 is an important cortical visceral motor center holds across anatomical and functional studies in rodents, non-human primates and humans. This suggests that there are aspects of area 25’s function in physiological domains that are conserved across species and given the importance of autonomic activity in the generation of affect, it would not be unreasonable to expect some degree of similarity in the effects of area 25 manipulations on affective behavior. This may indeed be the case when comparing human, non-human primate and rodent findings with respect to reward processing. Area 25 activation in marmosets blunts appetitive anticipatory and motivational arousal, effects reversed by an acute dose of ketamine, thus mirroring the reduction in area 25 activity following successful treatment in patients suffering from treatment-resistant depression. IL activation in rats also tends to reduce rewarded responding. Nevertheless, caution is warranted when drawing comparisons between behavioral and autonomic correlates of anticipatory affect in non-human primates and rodents and subjective states in humans. Future studies in the clinic should include the measurement of additional physiological and behavioral outputs to facilitate translation.

Critical to overall progress in this field will be closer integration between studies across humans, monkeys and rodents, with monkeys acting to bridge the gap between rodents and humans. Especially important will be identifying under what circumstances area 25 is activated and determining the interaction between cognitive, physiological and behavioral functions associated with this region. From studies so far it can be seen that functional similarities between monkey area 25 and rodent IL are far from clear and in some cases appear opposite. As discussed above, these may be due, in part, to differences in the pharmacological tools used to induce activation and the duration of such effects, along with variations in the psychological/cognitive mechanisms engaged to perform a given task. However, it should also be borne in mind that whilst IL and area 25 are considered structurally homologous, the anatomical framework in which they reside is not. Primate area 25 is operating in concert with the highly developed dorsolateral (dlPFC), ventrolateral and frontopolar cortices, which are likely to be contributing to many of the tasks in which area 25 manipulations have been investigated. Thus, they may contribute to some of the differences observed between area 25 manipulations in primates and rodents. Given the vast expansion of PFC in primates it is more than likely that there has been an expansion and specialization of cognitive functions, in which the rudiments are instantiated in more generic processing modules within the rodent PFC.

Future studies in non-human primates should focus on these higher-order regions and begin to dissect out their interactions with area 25 to advance our understanding of the higher-order control of reward and threat-driven behaviors. By employing novel chemogenetic and optogenetic tools, the investigation of these specific non-human primate pathways descending into area 25 is now possible, alongside similar studies investigating selective efferents of area 25. This will be particularly important in establishing the multitude of functions that area 25 likely contributes to, given its extensive anatomical connectivity. 

An additional consideration is how best to translate these findings into the clinic. So far, most targeted area 25/IL manipulations in preclinical animal work involve transient activations or inactivations, but see [169]. However, disease states are associated with chronic, long-term changes in a distributed network of structures. For instance, it is a tonic elevation in area 25 that has been associated with depression, compared to acute changes associated with sadness induction in healthy controls [190]. At a cellular level, sustained changes in neural activity are more likely to cause receptor desensitization and endocytosis, together with functional and structural plasticity. At a circuit level, sustained alterations in activity of a single brain region are more likely to induce compensatory changes in other brain networks, which may be relevant to the disease phenotype. Finally, at a cognitive level, mental illnesses are associated with maladaptive learning over time, which further contributes to the behavioral, subjective and executive sequelae of these disorders. Thus, future studies should also determine the effects of chronic over-activity in area 25. These can be investigated using a variety of techniques that would permit such chronicity without the need for chronic brain interventions. These include the area 25-specific administration of siRNA targeting EAAT2 to inhibit glutamate reuptake or the chronic administration of DREADD (designer receptors exclusively activated by designer drugs) ligands using osmotic minipumps in animals with G_q_-coupled DREADDs targeting area 25 to increase neuronal excitability. Such studies should not only be undertaken in adulthood, but also at different stages of development, because disorders such as anxiety and depression very often have their onset during puberty and adolescence (for review see Young et al., this issue). Insight has already been gained into the functional development of rodent vmPFC (including IL) in relation to conditioned threat extinction (reviewed in Zimmermann et al, this Special issue), paving the way for similar studies of primate area 25. Indeed, the unique growth trajectory of area 25 was revealed in a recent study mapping marmoset structural growth trajectories of cortical and subcortical regions. Compared to neighboring prefrontal and ACC areas, area 25 displayed an early onset of grey matter decline (thought to reflect changes in synaptic plasticity, pruning and increased myelination) around the start of puberty which then continued throughout adolescence, with the greatest rate of decline not occurring until the end of adolescence [191]. This prolonged period of structural ‘change’ may make area 25 particularly vulnerable to external stressors across extended periods of development and may explain its prominent role in models of depression. As reviewed in Datta and Arnsten (this Special issue) prefrontal cellular circuits, as revealed by studies of the cellular mechanisms underlying working memory in dlPFC, appear particularly vulnerable to uncontrollable stress, effects that can be exacerbated during adolescence, but whether similar or different interactions are played out in area 25 during development are as yet unknown. 

## 5. Conclusions

Area 25 plays a key, causal role in physiological and behavioral changes that resemble symptoms of enhanced negative affect and anhedonia, constituting key features of major psychiatric disorders such as depression and anxiety. However, other regions within the vmPFC also play a role, and care must be taken not to equate area 25 with the vmPFC. Further characterization of behavioral and cognitive functions subserved specifically by area 25 in the non-diseased state will aid in delineating the importance of dysfunction within area 25 to the phenotype of these conditions. Work in non-human primates, whose vmPFC is more similar in its overall organization to humans than rodents, is critical to this effort. Success is dependent on far closer integration of studies across species and more readiness to acknowledge differences, as well as similarities, across those species.
